# Real-Time Robust Tracking for Motion Blur and Fast Motion via Correlation Filters

**DOI:** 10.3390/s16091443

**Published:** 2016-09-07

**Authors:** Lingyun Xu, Haibo Luo, Bin Hui, Zheng Chang

**Affiliations:** 1 Key Laboratory of Opto-Electronic Information Processing, Chinese Academy of Sciences, Shenyang 110016, China; luohb@sia.cn (H.L.); huibin@sia.cn (B.H.); ChangZheng@sia.cn (C.Z.); 2 Shenyang Institute of Automation, Chinese Academy of Sciences, Shenyang 110016, China; 3 University of Chinese Academy of Science, Beijing 100049, China

**Keywords:** visual tracking, motion blur, fast motion, correlation filter

## Abstract

Visual tracking has extensive applications in intelligent monitoring and guidance systems. Among state-of-the-art tracking algorithms, Correlation Filter methods perform favorably in robustness, accuracy and speed. However, it also has shortcomings when dealing with pervasive target scale variation, motion blur and fast motion. In this paper we proposed a new real-time robust scheme based on Kernelized Correlation Filter (KCF) to significantly improve performance on motion blur and fast motion. By fusing KCF and STC trackers, our algorithm also solve the estimation of scale variation in many scenarios. We theoretically analyze the problem for CFs towards motions and utilize the point sharpness function of the target patch to evaluate the motion state of target. Then we set up an efficient scheme to handle the motion and scale variation without much time consuming. Our algorithm preserves the properties of KCF besides the ability to handle special scenarios. In the end extensive experimental results on benchmark of VOT datasets show our algorithm performs advantageously competed with the top-rank trackers.

## 1. Introduction

Visual object tracking plays an active role in military guidance, robot navigation, medical image processing, virtual augment reality and many other applications. Nevertheless, an efficient tracker faces combined challenges due to varying circumstances. First, the scenarios may suffer from illumination variations, changing camera postures and background clutters. Second, the appearance of target itself may have variations on scale, aspect-ratio, color or deformation in non-rigid object. Moreover, the typical low speed of camera and high speed of target make fast motion a common problem in visual tracking. Meanwhile, in a considerable number of scenarios, critical feature extraction and classical tracking approaches fail affected by motion blur due to the high relative speed [1].

Many research efforts have been focused on building appearance models of targets to estimate the variation of target in the past decades. In a sort of way conventional algorithms extract features from the target patch and evaluate the affine matrix or the consistency to reap the new position or probability distribution of target. Say, NCC [2] is a typical tracking algorithm using images matching which searches the cross-correlation values of the template image and candidate patches to predict the target position with best score. To improve the search strategy of target motion, Akbariet et al. [3] integrate Particle Swarm Optimization and Kalman filter into a framework for tracking. Comanicu D et al. [4] apply mean shift to build a fast search scheme for optimization using grey features. Lucas and Kanade [5] evaluate the Optical Flow between consecutive frames by minimizing the constrained energy function caused by three hypotheses about the consistence of pixels and voxels. Such traditional algorithms almost have no considerations on variations of the target appearance model.

Image sparse representation is hot research recently for it has a merit of representing signal flexibly and can avoid noise effects and appearance changes to some extent when maintaining features of targets. Sparse representation has been applied to tracking task in [6], and later be speeded up in [7]. Although it improves the speed for the task, sparse representation always consumes large computation that may not meet the real-time request in applications. There are also useful algorithms whose starting point is the Bayesian networks frame. In [8], conditional random filed is exploited to evaluate the matching score of target. However, these methods also suffer from the larger variations of target or scenarios, including applications with motion blur and fast motion.

Another view of tracking is to take the task as a binary classification between target foreground and the background. Kalal [9] takes the tracking task as a tracking, detection and learning(TLD) problem, which makes full use of the target and motion information. This mechanism has been proved to be effective and flexible. If the location information of object in previous frame is individually reaped to estimate the current target, the appearance model may accumulate errors and once the object disappears, the target will fail to be tracked permanently. The updating classifier detector by new samples coming in adjusts the appearance model in a more reliable way. Also inspired by this idea, (Struck) [10] introduces a clear output space and proposes a structure output prediction based adaptive visual tracking framework. The TLD tracker and Struck tracker outperform most of the traditional trackers in public test especially when the appearance model of a target changes heavily or gets lost, but they also have difficulties when handling the fast or blur motions of a target and always assume large computation.

Among the state-of-the-art methods, recently the algorithms based on correlation filters shows its advantages both in accuracy and robustness. Through introducing frequency domain transformation, Henriques et al. [11] consider the detection as a binary kernel ridge regression problem. As a tracking-by-detection tracker, the processing speed of Kernelized Correlation Filter (KCF) fantastically is fantastically dozens of times that of TLD or Struck. The multi-channel features and the approach to integrate them together are applied in KCF to build an insensitive stronger classifier for illustration variation and appearance model variation. In this way, the tracker is easier to understand and can be supported by richer powerful features e.g., CNN features and textural features, rather than just using the raw greyscale pixels. It is more efficient than the model with just single feature and has the potential to be widely used for real applications. However, there still remain many challenges, say, the scale variation, majority occlusion motion blur and fast motion. 

In this paper, we present a new scheme to deal with fast motion and the motion blur caused by fast motion without deblurring the image. Our algorithm achieves much higher accuracy and robustness competing with the top-rank trackers. Our main contributions can be concluded as follows: (1) analyze the property of frequency-domain feature for blurred image; (2) utilize sharpness point function to evaluate the motion of target; (3) build an active and search scheme on kernelized correlation filter for fast and robust tracking of scenarios with fast and/or blur motion. Our method has achieved excellent performance competing with the current rank-top trackers on extensive experiments; in addition, our method reaches a high speed even in worst scenarios.

## 2. Related Works

In this part, the related works is discussed for three problems: (1) correlation filter based tracking; (2) blur motion and fast motion handling; (3) Scale variation handling.

### 2.1. Correlation Filter Based Tracking

Heads from signal processing, correlation filter (CF) recently gives great rise to interests in image detection and classification. In signal processing, the cross correlation of signals assesses the similarity between them and reaches the extreme value when the two functions for signals are the same. This property can be utilized to evaluate the similarity of two image patches. Adopting the principle of achieving the maximum cross correlation response between its model and candidate patch, the Minimum Output Sum of Squared Error (MOSSE) filter is proposed by David S. Bolme et al. [12] to reap the appearance model of target. Instead of computing the inverse matrix as many algorithms do, MOSSE only requires divide operation in frequency domain, which makes its robustness and speed both compelling. Hamed et al. [13] ameliorate the way of selecting training patches to dramatically reduce both the boundary effects and expensive sampling computations for MOSSE. 

Despite the advances of MOSSE, it has prime problems in two aspects: (1) the way of sampling consumes much computation; (2) as a classifier, it could be stronger and more flexible. Introducing the kernel trick for classifier improvement and the cycle shift technique for fast sampling, Henriques et al. [14] take the training model as a ridge regression question and then give the corresponding quick solution. To get better interpretability and higher adaptability for diverse scenarios, Henriques et al. also introduce multi-channel HOG features and further create a scheme to integrate selected multiple features into the tracking framework. The KCF tracker has made great improvement both in accuracy and processing speed, meanwhile it has been paid so much attention that recently corresponding researchers devote to fixing it in different aspects. For better feature detection, literature [15] discusses how various convolution neural network features affect the accuracy of CFs, and draws a conclusion that the feature in the first level wins. In [16], a part-based model strategy is applied to cope with deformation of target. A scale adaptive Kernelized Correlation Filter (SAMF) [17] relieves the problem of scale estimation for target by applying a Laplacian Pyramid with a pool of 7 different scales. Some other peer trackers combine long-term tracking with KCF. For instance, Long-term Correlation Tracking [18] implements an active scheme to utilize random ferns for re-detection and Muster [19] build a biologically inspired system for cooperation of CF short term tracking and long term tracking. However, there has not been an effective scheme to handle with fast motion and motion blur under this tracking frame.

### 2.2. Blur Motion and Fast Motion Handling

Fast motion and motion blur are two prominent problems in tracking system, which have also been pervasively encountered in real applications, e.g., moving objects in large scenes, shaking camera. The problems for blur motion and fast motion include: (1) fast motion between camera and target makes estimating motion parameters more difficult even in consecutive frames; (2) motion blur brings about slightly or greatly negative effects on the performance of the traditional feature based detectors. An spontaneous idea to settle these problems is to deblur the blurred candidate patch and then apply original tracking methods. Although this could be theoretically successful, the current algorithms for deblurring image consume large amount of time which makes real-time tracking improbable. Blur-driven Tracker (BLUT) [20] builds a set of blurred images with different parameters as models for sparse representation. Since the blur motion model could be formulated as the convolution of a Point Spread Function (PSF) and the original image, Dai and Wu [21] propose a method to estimate the PSF to track the blurred target. In [22], a matching score function caused by the cost function between blurred image and model is introduced to search the target region.

Our algorithm avoids estimating the parameters of blur model and adaptively handles the problem with slightly or greatly blurred or fast motion. Meanwhile, we have not a higher computational complexity of our method for visual sequences with from barely to greatly fast or blurred motion.

### 2.3. Scale Variation Handling

Since the original KCF algorithm has no scheme for scale variation estimation, in the recent two years certain improved KCF algorithms focus their efforts on this problem. As discussed above, SAMF exploits Laplacian Pyramid of multi levels on the candidate patches and updates the model by resizing the detected target to a certain scale. Multi-kernel Correlation Filter [23] advices to apply the frequency peak sidelobe ratio (PSR) of target patch for estimating its scale. Spatio-temporal Context tracker (STC) [24] observes that the variation of scale affects the score of the estimated target position in Bayesian confidence map. For fast and accurate scale estimation, our method improves the scale variation of STC via considering the variation of confidence the motion model brings about.

## 3. Approach

We propose a new method to combine KCF and STC for scale estimation, and practice our new scheme for motion blur and fast motion testing. In the first place, the KCF algorithm is re-formulated and fixed with scale estimation; secondly the foundation of our algorithm is theoretically analyzed. At the end, the detailed steps of the algorithm for fast and blur motions are given.

### 3.1. Re-Formulate Kernelized Correlation Filter Tracking with Scale Handling

The tracking frame of Kernelized Correlation Filter, like other correlation filter algorithms, is inspired by MOSSE tracker. Now the scheme of KCF tracker is specified. At first, let’s consider the ridge regression problem: given the training data set and their labels {(**x***_i_*, *y_i_*)}*_i_*_=1~*n*_, function *f*(**z**) = **w*^T^*z** could be found to satisfies the equation below,

(1) $$\underset{w}{\arg\min}\sum_{i}{\left(f(x_{i})-y_{i}\right)}^{2}+\lambda {\left\|w\right\|}^{2}$$

where λ is the penalty coefficient of regularization item, preventing the over-fitting phenomenon. Our aim is to train a model **w** to best satisfy the training samples based on the Linear Least Squares criteria. Here is the explicit solution for Equation (1) in complex fields:

(2) $$w={\left(X^{H}X+\lambda I\right)}^{-1}X^{H}y$$

where $X^{H}={\left(X^{*}\right)}^{T}$ is the complex conjugate matrix of X. Introducing “kernel trick” to support a richer model, the model can be re-formulated as

(3) $$\underset{\alpha }{\arg\min}{\left\|K\alpha -y\right\|}_{2}^{2}+\alpha K\alpha$$

where **K** is the kernel matrix with dot-product in Hilbert space as its elements $\langle \varphi (x_{i}),\varphi (x_{j})\rangle =k(x_{i},x_{j})$. According to the relevant theory, the solution to the classical kernelized ridge regression can be given by:

(4) $$\alpha ={\left(K+\lambda I\right)}^{-1}y$$

Since this solution involves the matrix inverse operation, the computational complexity could increase fast. Luckily, Henriques et al. have proved in [11] that if ***K*** is a cyclic matrix and some certain conditions are met by ***K***, then the solution in frequency domain can be simplified as:

(5) $$\hat{\alpha }=\frac{\hat{y}}{{\hat{K}}^{xx}+\lambda }$$

Here ***K****^xx^* is the first row vector of the cyclic matrix ***K***. The hat symbol ˆ represents the Fourier transformation of a vector. For convenience, they also give three most typical kernel functions qualified for the theory, namely, Polynomial Kernel, Gaussian Kernel and Linear Kernel. In specially, when selecting a Linear Kernel, the problem is reduced to original regression problem without relative kernel trick correspondingly. Once securing or initializing the coming target patch *x*′, the kernel matrix in frequency domain is calculated:

(6) $$K^{xx\prime }={\left(F^{-1}(\hat{x}⊙\hat{x}\prime^{*})+a\right)}^{b}$$

(7) $$K^{xx\prime }=\exp(-\frac{1}{\delta^{2}}({\left\|x\right\|}^{2}+{\left\|x\prime \right\|}^{2}-2F^{-1}(\hat{x}⊙\hat{x}\prime^{*})))$$

Here Equation (6) is for Polynomial Kernel and Equation (7) is for Gaussian Kernel. ⊙ denotes element-wise multiplication and ∗ means the complex conjugate of a vector. The vector *x* represents the appearance model and in the first coming frame, *x* is initialized to *x*′.

The response for position **z** is then estimated by parameters **α** and **x**:

(8) $$\hat{f}(z)={\hat{K}}^{xz}⊙\hat{\alpha }$$

To catch the variations of target appearance model, the KCF tracker has its scheme to update its template with fixed learning rate *η* (set as 0.2 in our experiment):

(9) $$\left\{ \begin{array}{ll} ℱ{\left(\alpha \right)}^{t} & =(1-\eta )ℱ{\left(\alpha \right)}^{t-1}+\eta ℱ(\alpha )\\ {\hat{x}}^{t} & =(1-\eta ){\hat{x}}^{t-1}+\eta \hat{x} \end{array}\right.$$

Since KCF has no scheme to handle with scale variation, the target scale could be estimated by referring to the STC tracker. Unlike other discrete scale evaluation methods e.g., SAMF, the STC tracker has a simple but effective scheme to flexibly calculate the scale variation of target. Its principle starts from a statistical point that if other conditions remain unchanged, the score of Bayesian confidence map goes down descend as the scale gets bigger. In detail, the target scale at frame *t* is given by:

(10) $$s_{t}={\left(\frac{p({\left(x_{0}\right)}_{t})}{p({\left(x_{0}\right)}_{t-1})}\right)}^{\frac{1}{2}}$$

where *x*_0_ is the evaluated position of target and *p*(*x*_0_)*_t_* is the confidence in frame *t*, namely the score tracker acquires for position *x*_0_. For more reliable and stable estimation of target scale, the updating scheme for *s* contains an inertia item calculated by mean of {*s_i_}_i_*_=1:*t*_:

(11) $$\left\{ \begin{array}{ll} \bar{s} & =(1/n)\sum_{i}s_{i}^{\prime }\\ s_{t+1} & =(1-\eta )s_{t}+\eta \bar{s} \end{array}\right.$$

where *η* is again a fixed learning rate. Above all is the basic idea and theoretical derivations for the KCF tracker with a scale evaluation scheme used in the STC tracker. The two methods are fused and summarized in Algorithm 1.
**Algorithm 1.** The KCF tracker with target scale estimation**Inputs:** Template ***x***, target position *x*_0_ initialized by the given ground truth in the first frame;Initial scale *s* = 1;Corresponding image sequence for tracking;**Outputs:** Estimated target position *x*_0_′ and estimated target scale *s*′ for each frame;Updated ***x***′ and model coefficient ***α***′ at each time;1:Selection: Select kernel function and features type;2:Initialize the model coefficient ***α*** with Equation (5);3:**for** *every coming frame* **do**4:Get the candidate image patch by {*x*, *s*} and resize the patch to the same size as ***x*** via bilinear interpolation;5:Calculate the kernel function ***K****^xx^*^′^ with Equation (6) or (7) according to the selected kernel;6:Calculate the response *f*(***z***) with Equation (8);7:Acquire the position of the maximum response *x*_0_′ and new size *s*′ with Equations (10) and (11);8:Update the template by Equations (5) and (9);9:**end for**10:**Return** {*x*′, ***α***′, *s*′}.

### 3.2. Analysis of Motion Blur and Fast Motion in Frequency Domain

**Point** **Spread** **Function.** The degradation model and its influence of motion blur on our tracker are analyzed in this section. If there is relative motion between the photographic apparatus and the object in exposal moment, then the image captured from apparatus always gets blurred. In visual tracking, motion blur caused by fast relative motion between the target and background often makes trouble for building the appearance model of target. We should have a big concern with it. Generally, PSF is employed to approximate the motion model for linear and shift invariant system. Assume the original image is *f*(*x*, *y*), the blurred image could be given by:

(12) g(x,y)=f(x,y)⊗h(x,y)+n(x,y)

where *h*(*x*, *y*) is the PSF and *n*(*x*, *y*) is the additive noise. ⊗ denotes function convolution. A motion vector could be disassembled into resultant movement concise of motions in vertical and horizontal directions. It’s noticeable that the motion between two consecutive frames is so slight compare to other motions that the motion can be regarded as uniform motion. For convenience, let’s consider the variation in horizontal direction first. The PSF for horizontal uniform linear motion can be written as:

(13) $$h(x,y)=\left\{ \begin{array}{ll} 1/L & ,\left|x\right|\leq L,y=0\\ 0 & ,else \end{array}\right.$$

To get information about the blurred image in frequency domain, the function *h*(*x*, *y*) in Equation (12) is replaced by Equation (13) and its frequency format is derived via Fourier transform:

(14) $$\begin{array}{l} G(u,v)=F(u,v)⊙H(u,v)\\ =F(u,v)\cdot \iint h(x,y)\exp(-j2\pi (ux+vy))dxdy\\ =F(u,v)\cdot \int_{0}^{L}\frac{1}{L}\exp(-j2\pi ux)dx\\ =F(u,v)\cdot \frac{\sin(\pi uL)}{\pi uL}\exp(-j\pi uL) \end{array}$$

**Figure 1:**
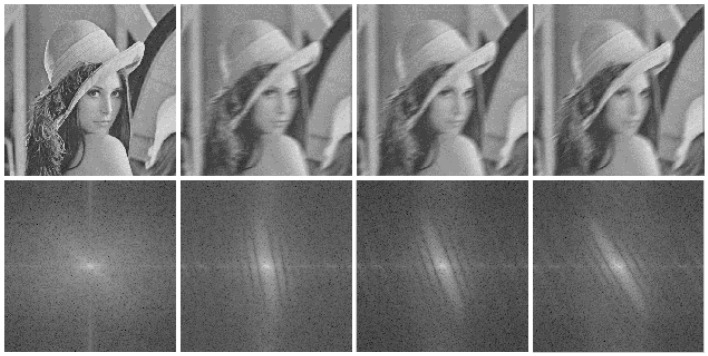
Figures from left to right are: original image with no motion, 20°, 40°, 60° movement in the horizontal direction. The figures blew are their corresponding spectrum.

Observing Equation (14), the spectrum for *g*(*x*, *y*) is concise of vertical parallel stripes. In a more general sense, the motion vector can be decomposed to two vectors in horizontal and vertical directions respectively. The principle for uniform motion in vertical direction can be deduced in the similar way. Actually, it can summarize that the direction of the target motion is orthogonal to the direction of parallel stripes in frequency spectrum. Some examples are listed in Figure 1. The motion between two neighboring frames could be estimated as uniform motion for the mistiming is limited. In our algorithm, the PSF of blur motion is not been estimated. Instead, the direction *θ* for the motion is calculated and utilized to determine the distance between target positions of two frames to avoid interference objects nearby. This information is abundant for target estimation in our tracker.

**Radon** **Transformation.** If the black stripes in spectrum are considered as lines, the position and direction of the dark lines could be evaluated via radon transformation. Once the directions of these dark lines are calculated, the motion direction is obtained by rotating 90°. By calculating the line integral in specified direction, Radon transformation figure out projection of a matrix in the corresponding direction. The radon transformation along angle α is defined as:

(15) $$I_{\alpha }(x\prime )=\int f(x\prime \cos\alpha -y\prime \sin\alpha ,x\prime \sin\alpha +y\prime \cos\alpha )dy\prime$$

As we could see, the response to *I_α_(x*′*)* hits the maximum when there is a long straight line in the project direction. In order to get the movement direction, radon transformation of angle 0°~180° is made for the spectrum *G*(*u*, *v*) and acquire the vector combined with every maximum value of each angle. Then these maximum value are united together to get a curve with direction varying. The maximum value of this curve is respected to the motion direction. 

**The Motion Model in Frequency Domain.** Although motion blur is introduced by relative fast motion, the target may have a high speed for movement without motion blur. In this part, the motion model is discussed for these scenarios to find the elusive target position. CF trackers always employ correlation functions in frequency domain to evaluate the similarity of two image patch. When two functions are equal to each other, namely the two image patches are the same, the value of cross-correlation function hits the maximum value. In signal processing, the cross-correlation function of two signals is written by:

(16) $$R_{xy}(l)=\underset{T\to \infty }{\lim}\frac{1}{T}\int_{0}^{T}x(t)y(t+l)dt$$

This function is always utilized to test the delay for signal. It can be seen that convolution between PSF of uniform motion and image will not change the maximum response position of the cross-correlation function. Moreover, if a signal is spread from point *A* to point *B*, *R_xy_* secures the peak value in the position of delay epoch. Determining the delay time can be utilized to measure the rate of movement of the object in engineering application, which also suggests us to estimate the movement of the target. The estimation formulations will be specify in the next section.

**The Image Blur Metric.** The question remained is how to reduce computation consuming. In this part the choice criteria is presented for image blur metric in our tracker and the chosen one. One compelling advantage of the KCF tracker is the high speed, hence we manage to improve the tracker without much computing. Since the computation is mainly on larger range searching, how to reduce the unnecessary searching should be accounted. If we could have an evaluation of the target’s motion, then we could lock the searching range when it’s less important. Here we introduce clarity evaluation function of motion blurred image to figure out the intensity of the target movement. Familiar metrics for image clarity are variance metric, autocorrelation-based metric, derivative-based metrics, frequency threshold metric, etc. What we need is one without reference or complex computing, perhaps not too sensitive to introduce a complex scheme for our tracker. In experiment we select JNB [25] metric as our clarity metric function. This metric is based on Sobel operation and can be employed to handle different contents in one image without much computational complexity that satisfies our requirement.

### 3.3. The Tracker

**Figure 2:**
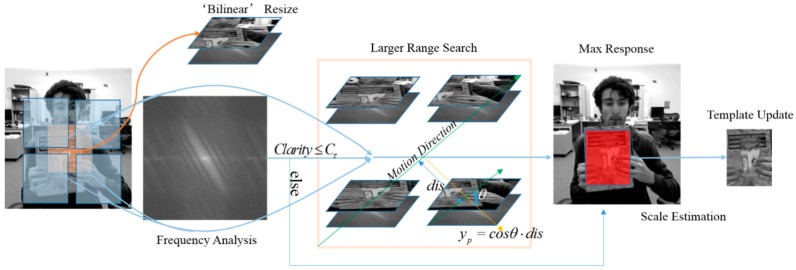
A brief illustration for our method.

In the former section the theoretical basis of the proposed algorithm is discussed, in this part we will specify our tracker. It’s important to note that all the parameters in our implementation are fixed and need no manual setting. There is a brief illustration for our tracker in Figure 2.

Denoting by *s* and *x*_0_ respectively the scale and position of target in the current frame, *s* and *x*_0_ from the target information is initialized at the first frame. Just as a slight clarification, all our experimental datasets can be find in Visual Tracking Benchmark [26], the information about target at the first frame is given as common protocol. At frame *t* + 1, the candidate image patch *x_s_* of scale *s* is searched nearby. Then this patch is resized to the same size as the template and its clarity value *c* is calculated via JNB metric. The clarity value is inversely proportional to the blur extent the target gets. For further computation, the FFT for *x_s_* is analyzed. 

Meanwhile, in frequency domain, Radon Transformation is made for spectrum of *x_s_* and figure out the estimated movement direction. Denoting the clarity values in the last frames by {*c_i_*}*_i_*_=1~*t*_, the procedure of selecting threshold *c_τ_* is inspired by Ostu threshold method [27], and the value in process is initialized by a fixed value. Specifically, the calculation of *c_τ_* is given by:

(17) $$\begin{array}{l} c_{l}=\sum_{c_{i}<c_{\tau }}c_{i}/card(\{c_{i}\}<c_{\tau })\\ c_{h}=\sum_{c_{i}>c_{\tau }}c_{i}/card(\{c_{i}\}>c_{\tau })\\ c_{\tau }=\arg\max\{c_{l}*c_{h}*{\left(c_{l}-c_{h}\right)}^{2}\} \end{array}$$

where *c_l_* and *c_h_* respectively denote the mean value of low and high clarity values. When the clarity value exceeds the adaptive threshold *c_τ_*, calculated by Equation (17), it indicates that the target tends to be blur free or slightly blurred. Hence, the maximum response is calculated for template in the original range and output the target position for next coming frame. Otherwise, the search range is enlarged according to the blur extent by a shifting L above, below, to the right and to the left in the current frame. Here we have:

(18) $$L=\lambda r(c_{t}+1)/(c+1)$$

where *λ* is a positive coefficient and we set *λ* = 2.25 in experiment. 

Notice that simply enlarging the searching range may introduce another problem for this tracker: it may take the similar but wrong target nearby as its aim. To solve this problem, consider the cityblock distance between the point *p* in subwindows and *x*_0_, and the calculated movement direction *θ*. All the responses of four subwindows are weighted by:

(19) $$\begin{array}{l} dis(p,x_{0})=\sum_{i=1}^{2}\left|p_{i}-x_{0_{i}}\right|\\ w_{p}=\mu dis(p,x_{0})\cdot \cos\theta \end{array}$$

where *μ* is a positive parameter that controls the weight of relatively far patches and is determined in experiment by target size. Here we approximate the similarity between real movement direction and estimated movement direction via the cosine function of *θ*. Then the position of maximum response is outputted as the current target position. At last, we estimate the scale variation for target and update the template by Equations (10) and (11). The procedure of our algorithm is shown in Algorithm 2.
**Algorithm 2.** The Blur KCF tracker**Inputs:** Template x, target position *x*_0_ initialized by the given ground truth in the first frame;Initial scale *s* = 1;Corresponding image sequence for tracking;**Outputs:** Estimated target position *x*_0_′ and estimated target scale *s*′ for each frame;Updated *x*′ and model coefficient *α*′ at each time;1:Selection: Select kernel function and features type;2:Initialize the model coefficient *α* with Equation (5);3:repeat;4:Calculate the kernel function *K_xx_*′ with Equation (6) or (7) according to the selected kernel;5:Calculate the response *f*(***z***) with Equation (8) and acquire the maximum response Rmax;6:Compute the Clarity value c of the candidate image patch via JNB metric;7:Find the threshold cτ with Equation (17); //Larger range search;8:If *c* ≤ *c_τ_* then;9:Make Radon Transformation for patch to figure out the target movement with Equation (15);10:Enlarge the search range with a shift from *x*_0_ with Equation (18) of four directions;11:for every new candidate image patch;12:Calculate the kernel function *K_xx_*′;13:Calculate the response *f*′(***z***) and acquire the maximum response *R*′;14:Weighted the response with Equation (19);15:If *R*′ > *R*_max_ then *R*_max_ = *R*′;16:end;17:end if;18:Acquire the position of the maximum response *x*_0_′ and new size *s*′ with Equations (10) and (11);19:Update the template by Equations (5) and (9);20:Until End of video sequences;21:Return {*x*′, *α*′, *s*′}.

## 4. Experiments and Results

### 4.1. Quantitative Evaluation and Speed Analysis

**Figure 3:**
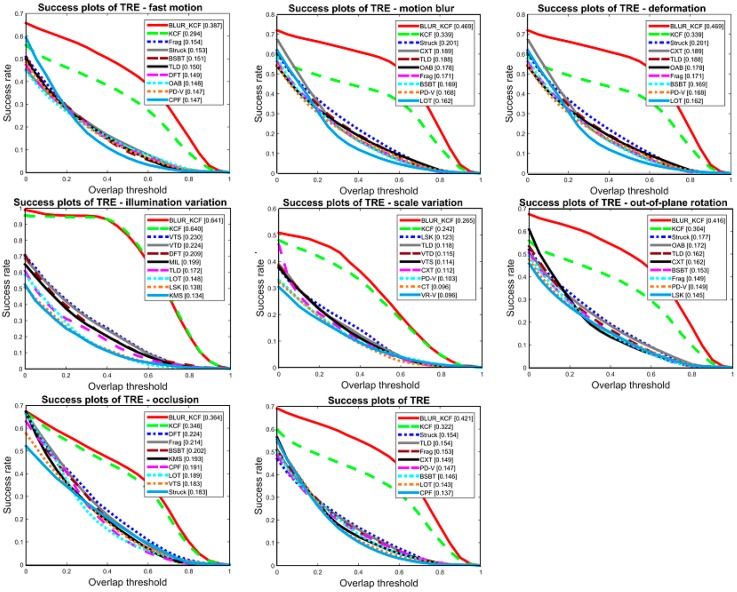
Overall performance in public datasets. These figures show the performance of trackers handing with fast motion, motion blur, deformation, illumination variation, scale variation, out-of-plane rotation and occlusion. The last figure shows the TRE success rate of these trackers under different threshold.

In this section our algorithm is mainly evaluated on a series of challenging sequences. We find out almost all the sequences with motion blur or/and fast motion in VOT benchmark and run our tracker on them, yet we did not find all these sequences’ running results for every state-of-art tracker. In order to compare our tracker with the top trackers, ten sequences from these sequences were selected with full results of all chosen trackers. Besides motion blur and fast motion, these video sequences are also suffered from other difficulties including occlusion, scale variation, illumination variation, in-plane/out-plane rotation, background clutter, etc. The trackers are run on these challenging sequences for general ability and special scenarios handling testing. These sequences are listed in Algorithm 1. As the protocol from [28], there are metrics from different perspective, namely One-Pass Evaluation (OPE), Temporal Robustness Evaluation (TRE) and Spatial Robustness Evaluation (SRE) are considered in our experiment. SRE evaluates the trackers by different initial annotation (slightly shifted from each other); TRE divides the sequences into 20 pieces and evaluates the trackers on different length of sequences; meanwhile, OPE evaluates trackers with other treatment. The overall performance is presented in Figure 3.

In order to evaluate our tracker in average performance, we compared our tracker with state-of-art trackers and list the top ten trackers in each metric. These 28 state-of-art trackers are from the code library of VOT, say, ASLA, BSBT, CPF, KCF, CXT, L1apg, Struck, MIL, SBT, TLD, ORIA, etc. In specify, we compare the center location error for each sequence with two other excellent trackers and the original KCF tracker. CXT, Struck outperform other 25 trackers over the dataset. The details are given in Table 1.

**Figure 4:**
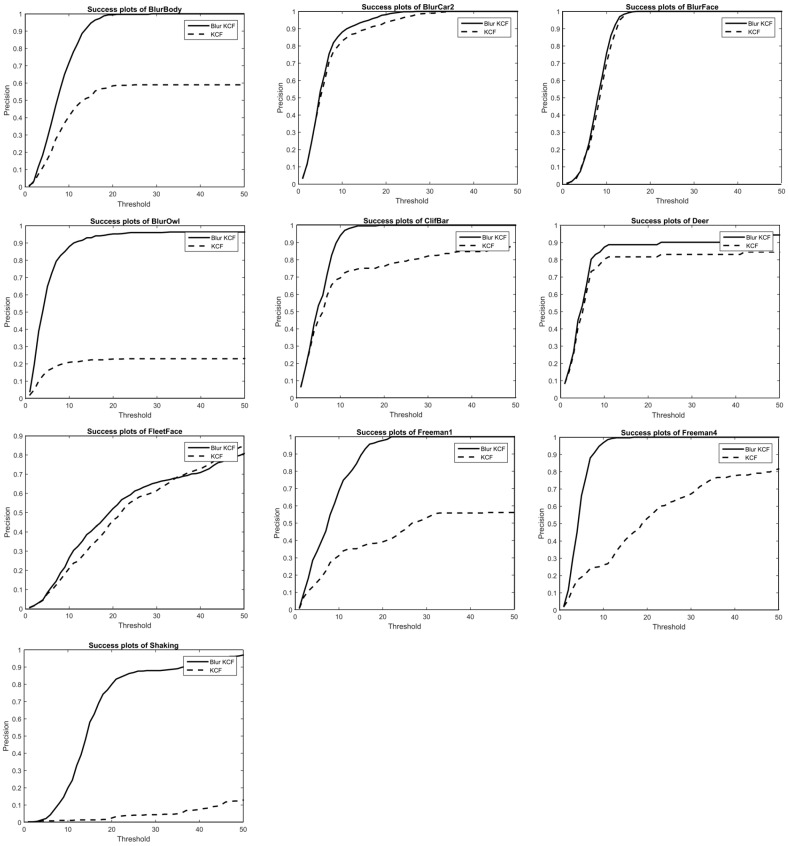
Precision plot in comparison with KCF.

These figures show the success rate plot comparison among trackers. In Figure 4, we plot the precision rate comparison between our algorithm and KCF tracker. Precision plot shows the percentage of frames whose location error measured in Euclidean distance is within the corresponding threshold in the *x*-axis; whereas the success plot shows the percentage of frames whose accuracy measured with $accuracy=\frac{\left|A_{t}^{P}\cap A_{t}^{G}\right|}{\left|A_{t}^{P}\cup A_{t}^{G}\right|}$ is within the overlap threshold. Additionally, our method has been tested with Matlab2014a on a PC, Intel corei5, 3.49 GHZ and 4 GB. Other improved KCF trackers like SAMF and part-based KCF take 5 times computation or more than KCF. Our tracker performs advantageously compared with these top trackers for sequences containing blur and fast motion that consumes not much computing.

### 4.2. Qualitative Evaluation

To evaluate the practical performance, the results of CXT, Struck, KCF trackers and our algorithm are also printed for qualitative evaluation. Take the BlurBody sequence for example, the camera is always shaking in this sequence as respect to the target boy. When there is a big shaking between the camera and target, the CXT and KCF trackers lost the position of target, Struck and our algorithm handle well this problem. This is because KCF utilize *dense sampling* that only search the candidate patch at the target position of last frame, leading to an error for search when the target moves fast.

**Figure 5:**
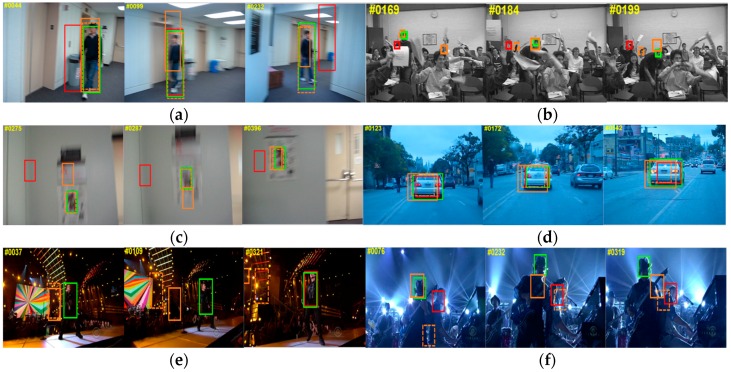
(**a**) BlurBody; (**b**) Freeman4; (**c**) BlurOwl; (**d**) BlurCar2; (**e**) Singer2; (**f**) Shaking. The results marked in orange, dashed orange, red and green are respectively from CXT, Struck, KCF and Ours.

As shown in Figure 5 below, the Struck tracker works well in the beginning frames of Freeman4 sequence but fails later by mistaking another similar person nearby as target. Our tracker has not influenced by this scenario since a scheme has been created to avoid the distraction from similar interfering substances by giving the relatively far candidate less weight. In BlurOwl sequence, almost every frame is subjected to varying degree of motion blur and many of them also involve fast motion. The KCF tracker lost target again when the motion is fast as stated and the CXT tracker also get lost when the candidate image patch gets blurred heavily for lacking of a scheme to handle blurred appearance model.

In the sequence of BlurCar2, Singer2, Shaking, besides motion blur and fast motion, the other main challenges for tracking are: scale variation, illustration variation, background clutter and illustration variation, respectively. From the figures in ‘BlurCar2’, we can see that benefits from the scale estimation strategy, the scale variation with the car was handled well when Struck and KCF get a shift for target. The results in sequence Singer2 show that our method keeps the virtue of KCF for background clutter. In sequence Singer2 and Shaking, accurate direction estimation in frequency domain helps the tracker to avoid the slight shift from target in illustration, the proposed tracker finish the tracking task well till the end when other trackers get lost.

## 5. Conclusions

In this paper we represent a new algorithm handling the motion blur and fast motion in visual tracking. We analyze the KCF tracker when it suffers from blur or fast motion, and improve the tracker without much computation. We consider the motion model between two frames to avoid inference of other similar objects. In this way our tracker keeps the advantages of original KCF tracker. Since the KCF tracker has no scheme to handle scale variation, we also fix the scale estimation problem by combining KCF and STC trackers together. At last, we compare our tracker with other 28 outstanding trackers in public tests. The results show our tracker performs advantageously compared with them.

## Figures and Tables

**Table 1:** Average center location error compared with other top trackers.

Title	Blur Body	Blur Car2	Blur Face	Blur Owl	Clif Bar	Deer	Fleetface	Freeman1	Freeman4	Shaking	Speed
CXT	25.94	26.8	19.29	57.33	33.08	19.99	57.3	20.41	67.46	157.39	9 fps
Struck	12.86	19.36	21.65	12.86	20.08	12.51	43.39	24.7	59.14	65.14	15 fps
KCF	64.12	6.81	8.36	92.2	36.7	21.16	26.37	94.88	27.11	112.5	**360 fps**
**Ours**	**11.95**	**5.82**	**8.01**	**8.88**	**6.04**	**9.46**	**26.37**	**8.06**	**4.5**	**17.5**	186 fps
